# Molecular detection of hemotropic mycoplasmas (hemoplasmas) in humans and dogs living on islands and the seashore mainland of Brazil: a One Health approach

**DOI:** 10.1186/s13071-025-07234-8

**Published:** 2026-01-27

**Authors:** Louise Bach Kmetiuk, Paul Shaw, Ashley Wallington, Jobin Jose Kattoor, Mathew Johnson, Rebecca P. Wilkes, Leandro Meneguelli Biondo, Rogério Giuffrida, Vamilton Alvares Santarém, Aaronson Ramathan Freitas, Ruana Renosto Delai, Claudia Turra Pimpão, Fabiano Borges Figueiredo, Joanne B. Messick, Andrea Pires dos Santos, Alexander Welker Biondo

**Affiliations:** 1 Department of Comparative Pathobiology, College of Veterinary Medicine, Purdue University, West Lafayette, IN USA; 2 Cell Biology Laboratory, Oswaldo Cruz Foundation, Curitiba, Paraná Brazil; 3 IDEXX Reference Laboratory, West Sacramento, CA USA; 4 Interdisciplinary Graduate Studies, University of British Columbia, Kelowna, BC V1V 1V7 Canada; 5 Graduate College in Animal Sciences, University of Western São Paulo (UNOESTE), Presidente Prudente, SP 19050-920 Brazil; 6 Graduate College in Veterinary Sciences, Federal University of Paraná (UFPR), Curitiba, PR 80035-050 Brazil; 7 Department of Animal Science, Pontifical Catholic University of Paraná (PUCPR), Curitiba, PR 80215-901 Brazil

**Keywords:** Zoonosis, One Health, Social vulnerability, Hemoparasites

## Abstract

**Background:**

Although *Mycoplasma* spp. infection has been recently detected in other vulnerable human populations (indigenous and *quilombola* communities) in Brazil, no study to date has focused on traditional oceanic island communities and their dogs. To address this research gap, we assessed *Mycoplasma* spp. infection in humans and dogs living on the mainland seashore and oceanic islands of southern Brazil.

**Methods:**

Humans from three oceanic islands and two coastal mainland municipalities of southern Brazil were sampled, and *Mycoplasma* spp. infection was determined using quantitative polymerase chain reaction (qPCR) (cycle threshold; Ct ≤ 34.4). Dog samples were collected and tested using the Canine Hemotropic Mycoplasma panel (Idexx Reference Laboratory, Sacramento, CA, USA). To ensure accurate results, samples were also subjected to targeted next-generation sequencing (tNGS), and results were used to construct phylogenetic trees. Epidemiological information was obtained to analyze associated risk factors.

**Results:**

A total of 19/304 (6.2%) individuals tested positive to hemoplasmas, with *Mycoplasma haemocanis* confirmed in 3/304 (1.0%) through 16S ribosomal RNA gene and targeted next-generation sequencing. In addition, 44/290 (15.2%) dogs were positive for hemoplasmas through qPCR testing, with 13/290 (4.5%) for *M. haemocanis*, 23/290 (7.9%) for *Candidatus* *Mycoplasma haematoparvum*, and 8/290 (2.8%) for both. Statistical analysis revealed an association between human positivity and gender and income range, and dog positivity was associated with male gender and access to forest areas.

**Conclusions:**

The concomitant human–dog *M. haemocanis* detected herein on oceanic islands together with results from previous reports on indigenous and *quilombola* communities, suggest that socially vulnerable populations have an increased exposure risk. Future studies should be conducted in other vulnerable populations worldwide to fully establish the extent of human–dog *Mycoplasma* spp. infection.

**Graphical Abstract:**

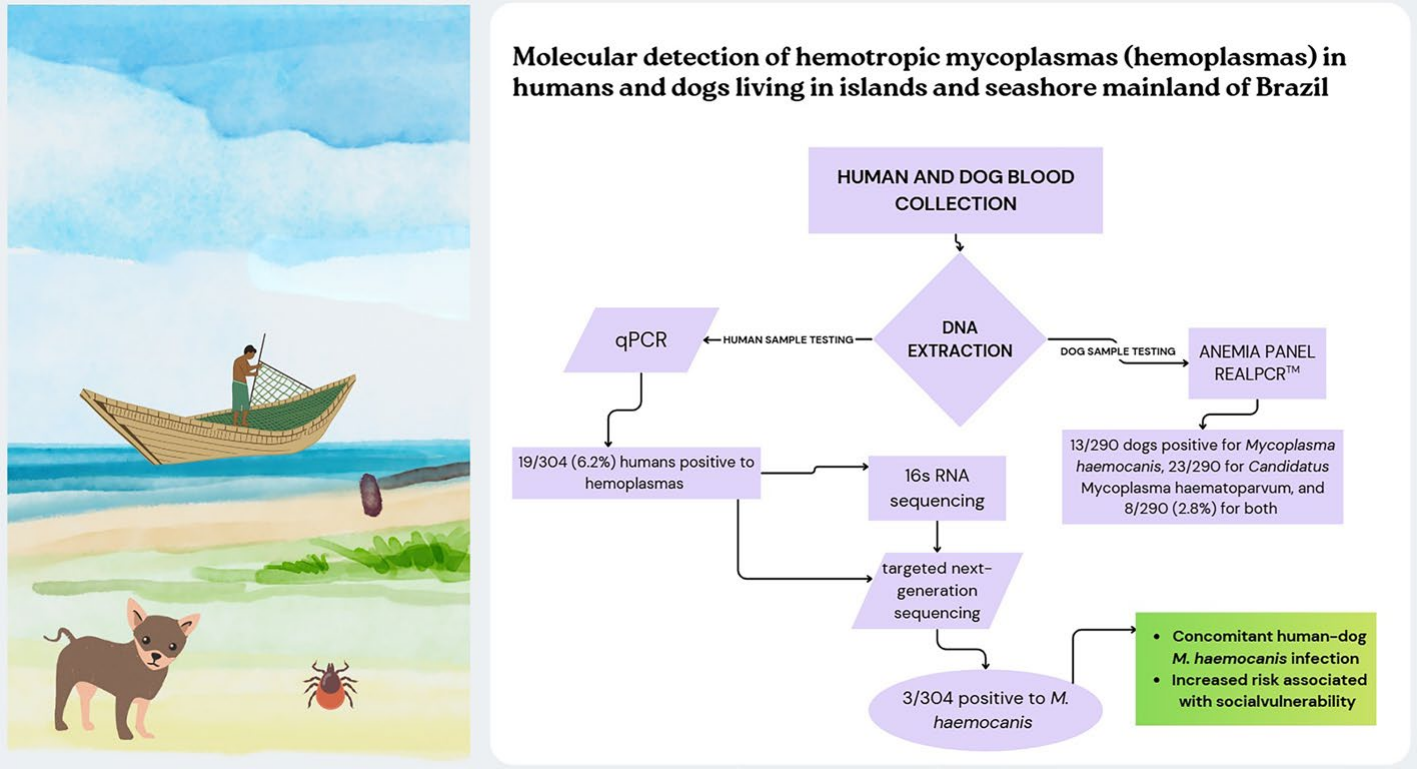

**Supplementary Information:**

The online version contains supplementary material available at 10.1186/s13071-025-07234-8.

## Background

Several species of hemotropic mycoplasma (hemoplasma) have been described in wildlife, domestic animals, and human beings, but the prevalence and transmission routes have not yet been determined [1]. Clinical signs of infection in humans and animals vary, but they usually include pyrexia, hemolytic anemia, lymphadenomegaly, and hepatosplenomegaly [2].

Previous reports have shown that humans can be infected with *Candidatus* Mycoplasma hominis, *Mycoplasma ovis*, *Mycoplasma haemofelis*, and *Mycoplasma suis* from sheep, cats, and pigs, respectively [3–6]. Infection is generally associated with occupational exposure, contact with animals, and natural areas, and veterinarians, travelers, farm workers, immunocompromised individuals, and hunters are mainly affected [3, 4, 7, 8]. In addition, *Mycoplasma* spp. has been detected in socially vulnerable individuals in close contact with positive dogs and potential vectors in natural areas [9, 10].

Island communities experience geographical isolation and limited access to health assistance, and they have a low responsiveness to public health challenges [11]. Appropriate sanitation, drinking water supply, and solid waste management practices are lacking within human and dog populations in island and seashore mainland areas within Brazil. Such populations also have contact with wildlife species in overlapped environmentally protected areas, and this may favor exposure to several zoonotic pathogens [12–17].

Island and seashore populations in southern Brazil are socially isolated from other populations, and they live closely with their dogs and other animals near natural areas that are home to wild animals potentially carrying vectors. However, no study to date has focused on human–dog infection with *Mycoplasma* spp. infection under such conditions. Therefore, we aimed to molecularly detect hemoplasmas in such populations.

## Methods

### Study design and area

A cross-sectional molecular survey was conducted. Sampling of humans and dogs living on three oceanic islands (Superagui Island, Mel Island, and Peças Island) and two seashore mainland municipalities of Paraná State (Guaraqueçaba and Pontal do Paraná), southern Brazil, was conducted from July 2019 to February 2020. The study areas were located within the most extended area of the Atlantic Forest biome of Brazil, overlapping different conservation units and protected areas [18]. Peças Island and Superagui Island comprise the Superagui National Park, which includes reefs, mangroves, and beaches and is home to endangered species [19]. Mel Island comprises two conservation units, an Ecological Station and a State Park, covering 81% and 12% of the total area, respectively [20]. Pontal do Paraná and Guaraqueçaba are two mainland cities that operate as access routes to these islands. Guaraqueçaba is a semi-isolated municipality located within three conservation units, and local access is difficult [19].

### Blood collection

After providing voluntary signed consent, human blood samples were collected by cephalic puncture conducted by certified nurses of the City Secretary of Health of each location. To obtain dog blood samples, the owner signed consent and completed epidemiological questionnaire, and samples were collected through jugular puncture by certified veterinarians. The blood samples were placed in ethylenediaminetetraacetic acid (EDTA) anticoagulant tubes, aliquoted, and stored at −80 °C until processing.

### Laboratory analysis

DNA was extracted from each human and dog blood sample using a commercially available kit (DNeasy Blood and Tissue Kit, QIAGEN, Hilden, Germany). Human samples were screened using a universal hemoplasma qPCR assay (SYBR™ Green PCR Master Mix, Applied Biosystems, Waltham, MA, USA) to detect hemoplasma 16S rDNA gene (∼ 200 bp), as already described [21]. Briefly, forward primer SYBR for 5′-AGC AAT RCC ATG TGA ACG ATG AA-3′ and the two reverse primers 5′-TGG CAC ATA GTT TGC TGT CAC TT-3′ and SYBR_Rev2 5′-GCT GGC ACA TAG TTA GCT GTC ACT-3′ in a concentration of 0.3 mM each were used for the reaction. The reaction mixture comprised 12.5 μL of 2 × SYBR green PCR master mix, 0.75 μL of each primer, 5 μL of DNA template, and 5 μL of RNAse-free water to entire volume of 25 μL. A previously established temperature was employed [22]. Two stored samples from dogs naturally infected with *Mycoplasma haemocanis* (GenBank accession no. CP003199) and two swine samples with *Mycoplasma suis* (GenBank accession no. ADWK01000001) were included as positive controls, and ultrapure water was used as the negative control. Positive samples were retested in triplicate and considered positive when the cycle threshold (Ct) was ≤ 34.4 [21]. The qPCR-positive samples were subjected to conventional PCR for nearly the entire 16S rRNA gene, using a commercial PCR mix (GoTaq Colorless Master Mix; Promega Corporation, Madison, WI, USA). The conventional PCR (cPCR) assay was conducted using a commercial mix (GoTaq Colorless Master Mix; Promega Corporation, Madison, WI, USA) following the manufacturer’s protocol. The cPCR proceeded using the forward primer 16S_HAEMOforw: 5′-GGC CCA TAT TCC T(AG)C GGG AAG-3′ and the reverse primer 16S_HAEMOrev: 5′-AC(AG) GGA TTA CTA GTG ATT CCA-3′, to amplify a DNA fragment of ∼966 bp. A total of 12.5 μL Master Mix, 1 μL of each primer (10 mM), 5 μL of nuclease-free water, and 5 μL of DNA sample were used. The thermoprofile was conducted using enzyme activation at 95 °C for 5 min, followed by 40 cycles of denaturation at 95 °C for 30 s, annealing at 55 °C for 1 min, and extension at 72 °C for 1 min, followed by one cycle of final extension at 72 °C for 5 min. A DNA sample of *Mycoplasma haemocanis* (GenBank accession no. CP003199), not previously detected in humans, was used as a positive PCR control, and nuclease-free water was used as the negative control. Amplicons with an expected DNA band size of approximately 1500 bp were purified using a PCR Purification Kit (QIAquick, QIAGEN). All positive samples were sequenced using the WideSeq method at the Purdue University Genomics Core Facility [23]. To ensure accurate results, samples were subjected to targeted next-generation sequencing (tNGS) as previously described [24] at the Animal Disease Diagnostic Laboratory (ADDL), which is the official diagnostic laboratory for the State of Indiana, USA.

Sequences obtained for this project were submitted to GenBank, and these sequences were highlighted in the tree with their accession numbers. Additional sequences were obtained from GenBank (including *Candidatus* Mycoplasma spp., which were considered an outgroup) for comparison. Alignment was performed using MUSCLE in MEGA 12 [25]. The best DNA model for the tree was determined using Model Selection in MEGA 12. The phylogeny was inferred using the maximum likelihood method and the Hasegawa–Kishino–Yano model of nucleotide substitutions. The tree with the highest likelihood is shown, and the percentage of replicate trees in which the associated taxa clustered together (1000 replicates) is shown next to the branches. Clades ≥ 70 were considered significant. These analyses were conducted using MEGA 12.

To detect *M. haemocanis* and *Candidatus* M. haematoparvum, dog blood samples were tested at IDEXX Reference Laboratory, Sacramento, California, USA, using the Anemia Panel RealPCR™ Panel–Canine with Anemia Panel Lab 4Dx Plus Test–Canine (2907) (proprietary information, Idexx Reference Laboratory, Sacramento, CA, USA) The Anemia Panel RealPCR™ Panel-Canine provides positive or negative results for *Mycoplasma haemocanis*, *Candidatus* M. haematoparvum, and can detect other pathogens including *Anaplasma* spp., *Babesia* spp., *Hepatozoon* spp., *Leptospira* spp., *Rickettsia* spp., and *Ehrlichia* spp. [26]. Interferences for PCR test included poor sample quality, an aged sample, or recent antimicrobial therapy. The Anemia Panel Lab 4Dx provides positive or negative results for *Dirofilaria immitis*, *Ehrlichia canis*, *Ehrlichia ewingii*, *Anaplasma phagocytophilum*, *Anaplasma platys*, and *Borrelia burgdorferi* [26].

### Statistical analysis

The overall and species-specific prevalence of *Mycoplasma* spp. was estimated with 95% confidence intervals (CI 95%). Univariate analyses were performed using Pearson’s chi-squared test or Fisher’s exact test to assess associations between a positive result for a dog and potential risk factors, including location (Guaraqueçaba, Peças Island, Mel Island, Pontal do Paraná, and Superagui), sex (female or male), mixed-breed status (yes or no), domicile (yes or no), access to the beach (yes or no), forest entry (yes or no), presence of ectoparasites (yes or no), use of flea and tick control (yes or no), and age category (puppy/juvenile: 0–3 years; young adult: 4–6 years; mature adult: 7–10 years; senior: ≥ 11 years).

The potential risk factors for human *Mycoplasma* spp. infection included locality (continental coast, island), age group in years (18–34, 35–43, 44–56, 56–93), gender (female, male), education level (higher education or more, completed secondary school, completed primary school, illiterate or not completed primary school), income (less than 1 minimum wage, 1–3 minimum wages, more than 3 minimum wages), native status (yes or no), number of dogs in household (no dogs, 1–2 dogs, 3–16 dogs), and tick bite history (yes or no).

Variables with *P* < 0.2 on univariate analyses for dogs and humans were considered eligible for inclusion in a multivariate logistic regression model. A backward stepwise selection procedure was applied to identify the model with the optimal explanatory capacity, on the basis of minimization of the Akaike information criterion (AIC).

Dogs or humans for which there were missing or incomplete data were excluded from the logistic final model, as the backward stepwise selection procedure required complete datasets to reliably estimate coefficients and perform model comparisons. Odds ratios (ORs) and their corresponding 95% confidence intervals were calculated for both univariate and multivariate models.

Model calibration was assessed using the Hosmer–Lemeshow goodness-of-fit test [27], while the discriminatory performance was evaluated by the area under the receiver operating characteristic curve (AUC) calculated by DeLong method. Collinearity among predictors was assessed by calculating the variance inflation factor (VIF). All statistical analyses were conducted in the blorr R package and using R internal functions [28, 29]. For all analyses, *P* < 0.05 was considered statistically significant.

## Results

**Fig. 1:**
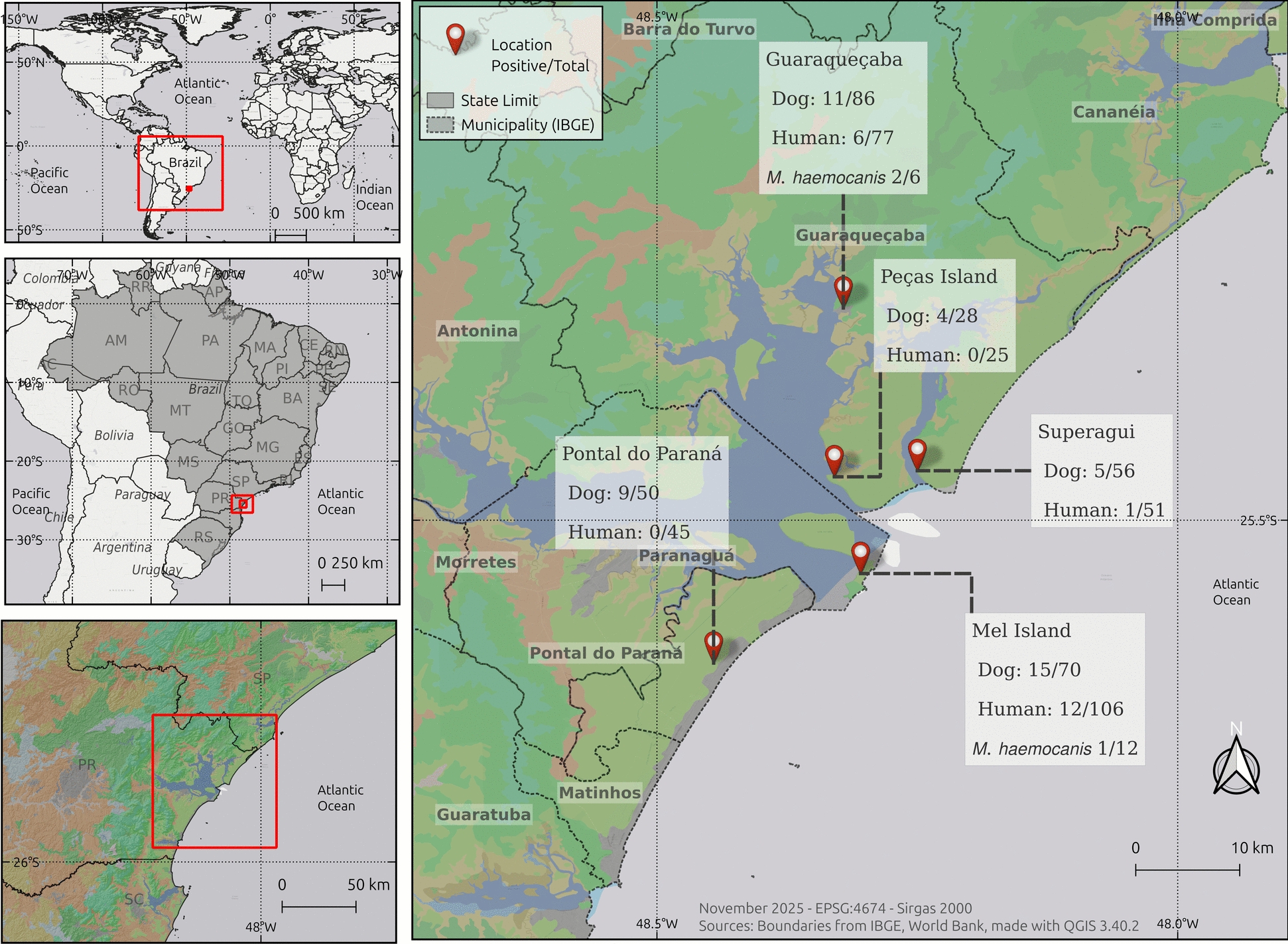
Positive samples to *Mycoplasma* spp. in seashore and islands of southern Brazil

**Fig. 2:**
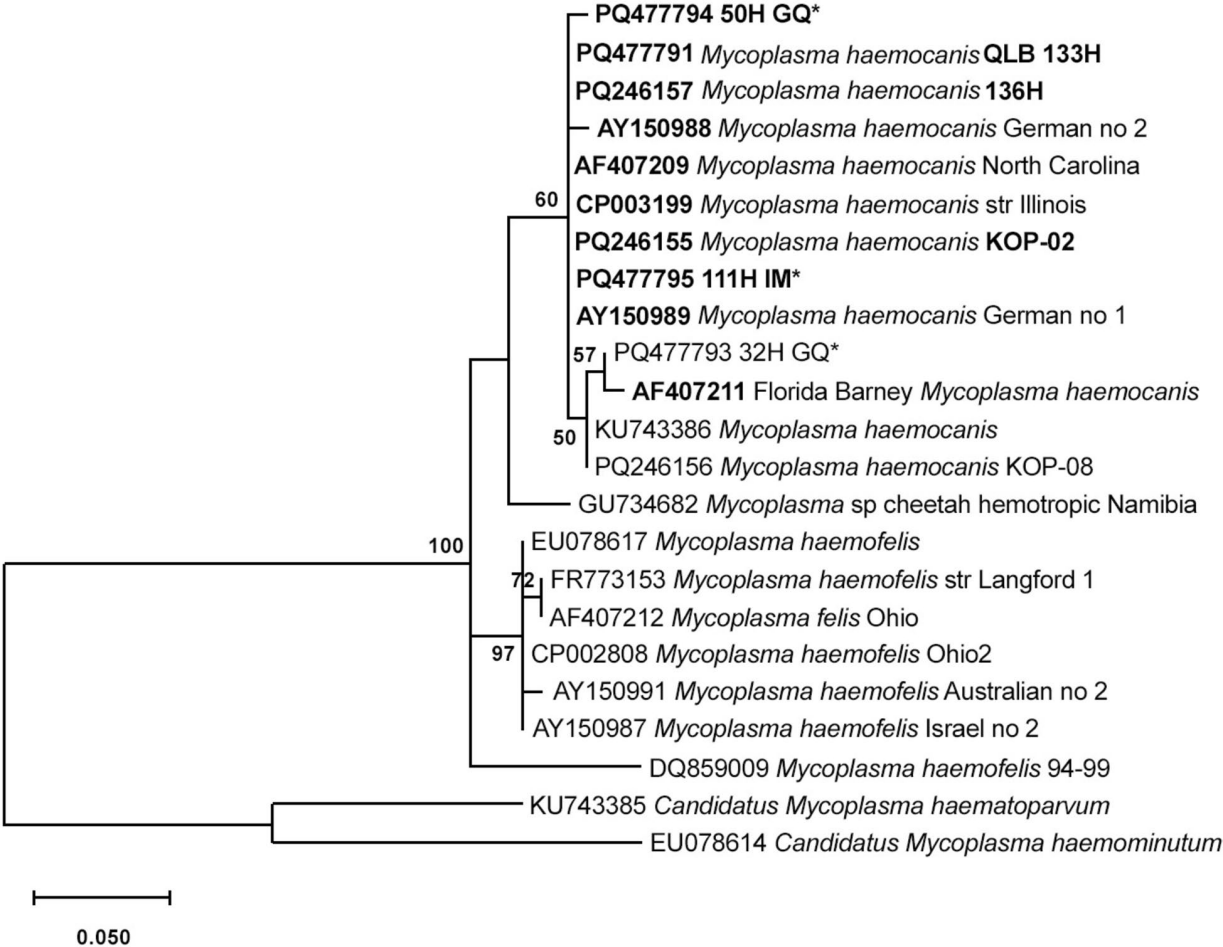
Phylogenetic tree of amplicon sequences obtained by tNGS of 16S rRNA gene sequences from positive human individuals

A total of 19/304 (6.25%; 95% CI: 4.04–9.55) humans were molecularly positive for hemoplasma infection by quantitative polymerase chain reaction (qPCR) (cycle threshold, Ct ≤ 34.4) (Fig. 1). The 16S ribosomal RNA gene sequencing confirmed *Mycoplasma heamocanis* infection in the 3 out of 304 (1.00%; 95% CI: 0.37–2.86), and *M. haemocanis* infection was also confirmed by tNGS in these three positive samples, two from Guaraqueçaba City and one from Mel Island (Fig. 2). Both RNase P and 16S gene sequences obtained from humans were submitted to GenBank (accession no. PQ477793, PQ477794, and PQ477795).

The overall prevalence of *Mycoplasma* spp. in dogs was 44/290 (15.2%; 95% CI: 11.50–19.75). Among the hemoplasma species, *M. haematoparvum* was the most frequently detected, with a prevalence of 23/290 (7.9%; 95% CI: 5.34–11.62), followed by *M. haemocanis* at 13/290 (4.5%; 95% CI: 2.64–7.52), and coinfection with both species was identified in 8/290 (2.8%; 95% CI: 1.40–5.35) dogs.

On univariate analysis, the variables with *P*-values < 0.2 considered for inclusion in the logistic regression model were sex, domicile, forest entry, presence of ectoparasites, and age categories. However, owing to missing data for the age variable, 27 dogs were excluded from the multivariate analysis. The final model was obtained after following the two-step backward stepwise procedure, retaining sex, forest entry, and age category as significant predictors. The results of the univariate and multivariate analyses are presented in Table 1.

**Table 1:** Associated risk factors for molecular *Mycoplasma* spp. detection in dogs of southern Brazil, Paraná (*n* = 290), by univariate and multivariate (logistic regression) statistical analyses

Variable	Serology		Statistical analysis			
	Positive	Negative				
	*n* (%)	*n* (%)	Univariate		Multivariate	
	44 (15.2)	246 (84.2)	OR (95% CI)	*p*	OR (95% CI)	*p*
Location				0.345		
Guaraqueçaba	11 (25.0)	75 (30.5)	Ref			
Peças Island	4 (9.09)	24 (9.76)	1.16 (0.29–3.82)			
Mel Island	15 (34.1)	55 (22.4)	1.85 (0.79–4.46)			
Pontal do Paraná	9 (20.5)	41 (16.7)	1.50 (0.55–3.96)			
Superagui	5 (11.4)	51 (20.7)	0.68 (0.20–2.02)			
Sex				0.048*		
Female	15 (34.1)	127 (51.6)	Ref		Ref	
Male	29 (65.9)	119 (48.4)	2.05 (1.06–4.12)		2.56 (1.19- 5.79)	0.019*
Mixed-breed status				0.477		
No	11 (25.0)	78 (31.7)	Ref			
Yes	33 (75.0)	168 (68.3)	1.38 (0.68–3.01)			
Domicile				0.050		
No	23 (52.3)	87 (35.4)	Ref			
Yes	21 (47.7)	159 (64.6)	0.50 (0.26–0.96)			
Access to the beach				0.514		
No	15 (34.1)	100 (40.7)	Ref			
Yes	29 (65.9)	146 (59.3)	1.32 (0.68–2.65)			
Forest entry				< 0.001*		
No	10 (22.7)	151 (61.4)	Ref		Ref	
Yes	34 (77.3)	95 (38.6)	5.32 (2.59–11.9)		5.96 (2.64–14.94)	< 0.001*
Presence of ectoparasites				0.183		
No	8 (18.2)	72 (29.3)	Ref			
Yes	36 (81.8)	174 (70.7)	1.83 (0.85–4.46)			
Use of flea and tick control				0.605		
No	14 (32.6)	67 (27.3)	Ref			
Yes	29 (67.4)	178 (72.7)	0.78 (0.39–1.61)			
Age group^a^				0.016		
Puppy/juvenile: 0–3 years	1 (2.70)	37 (16.4)	Ref			
Young adult: 4–6 years	5 (13.5)	32 (14.2)	5.13 (0.74–141)		3.60 (0.50–73.31)	0.268
Mature adult: 7–10 years	16 (43.2)	112 (49.6)	4.65 (0.90–115)		3.41(0.61–64.28)	0.254
Senior: ≥ 11 years	15 (40.5)	45 (19.9)	10.8 (2.01–269)		8.07 (1.40–153.99)	0.055

^a^ Missing data (27 dogs). Bold values indicate that the *p*-value lower than 0.05

The logistic regression model for dogs demonstrated good discriminative ability, with an area under the curve (AUC) of 0.777 (95% CI = 0.705–0.849), indicating that the model performed significantly better than chance in distinguishing between outcome categories. The Hosmer–Lemeshow goodness-of-fit test indicated no evidence of poor model fit (*χ*^2^ = 2.46, *df* = 8, *P* = 0.963). No significant VIF was detected for variables retained in final model (1.020 for sex, 1.0260 for forest entry, and 1.009 for age category).

The logistic regression coefficients and model parameters were gathered and are presented in Supplementary Tables S1 and S2 along with the receiver operating characteristic (ROC) curve in Supplementary Figs. S1 and S2.

The human variables with *P* < 0.2 included in the logistic model were gender and income range. However, owing to missing data for the income range, one human was excluded from the multivariate analysis. All predictors (gender and income range) were retained in the final model after conducting the backward selection procedure. The results of the univariate and multivariate analyses are presented in Table 2.

**Table 2:** Associated risk factors for *Mycoplasma* spp. positivity in humans from seashore mainland and islands of southern Brazil (*n* = 290), by univariate and multivariate (logistic regression) statistical analyses

Variable	Serology		Statistical analysis			
	Positive	Negative				
	*n* (%)	*n* (%)	Univariate		Multivariate	
	19 (6.2)	285 (93.7)	OR (95% CI)	*p*	OR (95% CI)	*p*
Location				0.587		
Seashore mainland	6 (31.6)	116 (40.7)	Ref			
Islands	13 (68.4)	169 (59.3)	1.47 (0.56–4.35)			
Age group (years)				0.217		
18–34	7 (36.8)	63 (22.1)	Ref			
35–43	3 (15.8)	70 (24.6)	0.40 (0.08–1.54)			
44–56	7 (36.8)	79 (27.7)	0.80 (0.26–2.50)			
56–93	2 (10.5)	73 (25.6)	0.26 (0.03–1.16)			
Gender				0.027*		
Female	7 (36.8)	185 (64.9)	Ref			Ref
Male	12 (63.2)	100 (35.1)	3.13 (1.21–8.79)		2.89 (1.11–8.08)	0.033
Education level				0.482		
Higher education or more	4 (21.1)	40 (14.0)	Ref			
Complete secondary school	8 (42.1)	131 (46.0)	0.60 (0.18–2.44)			
Complete primary school	5 (26.3)	53 (18.6)	0.94 (0.23–4.18)			
Illiterate or incomplete primary school	2 (10.5)	61 (21.4)	0.34 (0.04–1.95)			
Income^a^				0.015*		
Less than 1 minimum wage	8 (42.1)	44 (15.5)	Ref			
1–3 minimum wages	10 (52.6)	227 (79.9)	0.24 (0.09–0.68)		0.27 (0.10–0.74)	0.009
More than 3 minimum wages	1 (5.26)	13 (4.58)	0.47 (0.02–3.05)		0.46 (0.02–2.99)	0.492
Native status^a^				0.602		
No	11 (57.9)	189 (66.5)	Ref			
Yes	8 (42.1)	95 (33.5)	1.45 (0.54–3.75)			
Dog owner				0.523		
No dogs	2 (10.5)	29 (10.2)	Ref			
1–2 dogs	15 (78.9)	192 (67.4)	1.07 (0.28–7.65)			
3–16 dogs	2 (10.5)	64 (22.5)	0.46 (0.05–4.57)			
Tick bite history^a^				1.0		
No	13 (72.2)	200 (70.2)	Ref			
Yes	5 (27.8)	85 (29.8)	0.92 (0.28–2.56)			

^a^ Missing data (one human); Ref. = reference category. Bold values indicate that the *p*-value lower than 0.05

The Hosmer–Lemeshow goodness-of-fit test yielded *χ*^2^ = 0.334 (*df* = 1, *P* = 0.563). Owing to the small number of events, only two predictive groups were formed, which limited the test’s power. However, the nonsignificant result suggested no evidence of miscalibration. The model showed good discrimination, with an AUC of 0.718 (95% CI: 0.607–0.830), and negligible multicollinearity, as indicated by the low variance inflation factors for gender and income range (1.001 each).

## Discussion

To the authors’ knowledge, this is the first study of hemoplasma detection that concomitantly accessed humans and dogs living on oceanic islands and seashore mainland areas within natural areas and with close wildlife contact.

Previous studies conducted by our research group with this same population have shown that human and dog populations living in seashore areas are exposed to other vector borne diseases, including Brazilian spotted fever, Q fever, and Chagas disease [12, 16, 30]. In addition, the social vulnerability of this population also confirmed exposure to other zoonotic diseases such as *Toxoplasma gondii*, *Toxocara* spp., and *Brucella* spp. [14, 15, 17].

The disease prevalence in humans in this study (19/304, 6.2%) within seashore individuals was higher than that recorded of 23/644 (3.6%) indigenous individuals from ten communities in southern and southeastern Brazil [10] and 2/208 (5.8%) *quilombola* individuals from southern Brazil [9]. Another previous study conducted by our research group in rural and anthropized areas of southern Brazil showed no molecular positivity in 25 hunters, despite their close contact with 94/159 (59.1%) positive hunting dogs and 38/65 (58.5%) positive wild boars, along with tick exposure [22]. In addition, low human positivity was reported in 1/100 (1%) human from a rural settlement of southern Brazil [31]. Finally, the positivity determined in the current study was also higher than the 9/193 (4.6%) veterinarians, veterinary technicians, and partners of veterinary professionals who were in close contact with animals and vectors in the USA [8].

Some species of hemoplasma have been described in human case reports, including *Mycoplasma suis*, *Mycoplasma ovis*, *Mycoplasma haemofelis*, *Candidatus* M. haematoparvum, and recently *Candidatus* Mycoplasma haemohominis [4, 5, 7]. Human hemoplasma infections have been related to close contact with animals, immunocompromised conditions, and occupational exposure. In the present study, *M. haemocanis* was confirmed as the hemoplasma species involved in human infection of three individuals. Our research group previously diagnosed *M. haemocanis* in indigenous and *quilombola* individuals in Brazil [9, 10], and together with the present results, this reinforces the hypothesis that *M. haemocanis* occurs more often in individuals who are overexposed to and have close contact with infected dogs and who live under socially vulnerable conditions. Although *Candidatus* M. haematoparvum was not diagnosed in humans herein, this hemoplasma species was previously reported in a veterinarian (along with *Anaplasma platys* and *Bartonella henselae*) who presented with neurological signs such as seizure and headaches [7].

The positivity herein of 44/290 (15.2%) in dogs was similar to that of 19/100 (19.0%) dogs from *quilombola* communities in southern Brazil [9], 91/416 (21.9%) dogs from indigenous communities in southern and southeastern Brazil [10], and 78/437 (17.8%) household dogs from southeastern Brazil [32]. The results were lower than that of 94/159 (59.1%) hunting dogs [22] and 59/132 (44.7%) dogs from settlements, both from southern Brazil [22]. Although tick transmission has been suggested as the main transmission route [1], no statistical association was observed herein between *Mycoplasma* spp. positivity and the presence of vectors at the time of sampling. Hemoplasma transmission may occur in the absence of vectors by social contact or in transplacental form [33, 34], which may be exacerbated in island communities owing to the high number of unneutered dogs. The dogs in this study were infected by *Candidatus* M. haematoparvum (23/290; 7.9%) and *M. heamocanis* (13/290; 4.5%), the major hemoplasma species infecting dogs worldwide [6, 35–37]. In this study, male dogs were more likely to be positive for hemoplasmas, which has also been reported in dogs from indigenous communities in Brazil [10], hunting dogs in Italy [38], dogs from rural and urban areas in Chile [39], urban dogs in Iraq province [40], and dogs in northern Tanzania [41]. As already suggested, the higher exposure to hemoplasmas in male dogs herein may be the consequence of gender behavior due to aggressive territorial interactions and blood contact [42]. Access to forest areas was also found to be associated with hemoplasma infection in dogs in this study. The outdoor lifestyle and contact with wildlife can contribute to exposure to routes of possible hemoplasma transmission via ticks or through direct contact during fighting or wounds acquired during hunting [38, 43]. Similar to dogs, male humans were more likely to be positive to hemoplasmas in this study, and this could also be related to the gender behavior of hunting and associated access to forest areas. *Mycoplasma* spp. has been reported in both male and female patients [4, 5, 44]. Although female positivity has been reported in veterinarians, researchers, wildlife managers, and tourists, male positivity has been reported in other situations, such as in immunocompromised men, farmworkers, veterinarians, and insurance workers [4, 5, 8, 44]. Further populational studies should be conducted to better understand the association between hemoplasma infection and gender exposure, as well as with family income. In addition, further studies should compare Ct values and the results of full sequencing of positive samples between humans and dogs to determine whether the haplotypes are indeed the same.

The samples in this study were obtained and processed as follows: first, various professionals (nurses and veterinarians) obtained samples within the same workday. Human samples were separately processed, aliquoted, and stored (mostly owing to biosafety and handling regulations) away from dog samples. Second, DNA from human blood samples was extracted at the Oswaldo Cruz Foundation (Fiocruz), Brazil, and tested at the Clinical and Molecular Pathology Laboratory (CMPL) and the Animal Disease Diagnostic Laboratory (ADDL) in West Lafayette, IN, USA. Dog whole blood samples were shipped in dry ice from Brazil to USA for DNA extraction and molecular diagnostic testing at the Idexx Laboratories, Sacramento, CA. Third, each laboratory independently performed a different molecular approach method to detect and identify *Mycoplasma* species: qPCR at the CMPL and Wideseq sequencing tNGS at the ADDL, while both approaches were used at Purdue, and a vector-borne qPCR diagnostic panel was conducted at Idexx Laboratories. Thus, human–dog cross-contamination during sampling, sample handling, aliquoting, storage, shipment, DNA extraction, and molecular detection of nonviable DNA would have been very unlikely, if not impossible.

Finally, the subclinical or nonspecific clinical signs of hemoplasmosis in dogs and the lack of veterinarian assistance may contribute to the underdiagnosis of *Mycoplasma* spp. and its spillover in susceptible human populations. Therefore, to prevent the zoonotic transmission of diseases, veterinary preventive measures should be largely implemented, particularly in vulnerable populations, by employing anti-tick treatments, vaccines, and deworming. Although veterinary assistance in indigenous communities was established in 2025 by the Brazilian Ministry of Health as a consequence of our One Health research study [45], other vulnerable populations remain unassisted, including *quilombola*, inmates, the homeless, and traditional island and seashore communities.

## Conclusions

The concomitant human–dog *Mycoplasma haemocanis* infection detected in communities living on oceanic islands and in remote seashore areas in Brazil, together with our previous reports of indigenous and *quilombola* communities, may suggest that socially vulnerable populations are at an increased risk of exposure to the disease. Future studies should be conducted to fully establish human–dog *Mycoplasma* spp. infection rates in other vulnerable communities worldwide, and transmission routes and the clinical presentations of *M. haemocanis* in humans should be further investigated and established.

## Supplementary Information

Additional file1 (DOCX 50 kb)

## Data Availability

All data generated or analyzed during this study are included in this published article and its supplementary information files.
