# Supplementary material for: Molecular detection of hemotropic mycoplasmas (hemoplasmas) in humans and dogs living on islands and the seashore mainland of Brazil: a One Health approach
Source: Parasit Vectors. 2026 Jan 27;19:52. doi: 10.1186/s13071-025-07234-8 (PMC12849347; doi:10.1186/s13071-025-07234-8)
Supplement: Supplementary file 1 — Additional file1 (DOCX 50 kb) [file 13071_2025_7234_MOESM1_ESM.docx]

**Additional file 1**

**Table S1**. Logistic regression model results for factors associated with seropositivity.

| **Coefficient** | Estimate | Std. Error | z value | Pr(>\|z\|) |
| --- | --- | --- | --- | --- |
| **Intercept** | -4.817 | 1.119 | -4.305 | < 0.001 |
| **Sex** |  |  |  |  |
| Female | Reference |  |  |  |
| Male | 0.939 | 0.402 | 2.338 | 0.019 |
| **Forest entry** |  |  |  |  |
| No | Reference |  |  |  |
| Yes | 1.785 | 0.438 | 4.08 | < 0.001 |
| **Age group** |  |  |  |  |
| puppy/juvenile:0–3 years | Reference |  |  |  |
| young adult: 4–6 years | 1.279 | 1.155 | 1.107 | 0.268 |
| mature adult: 7–10 years | 1.227 | 1.076 | 1.14 | 0.254 |
| senior: ≥11 years | 2.089 | 1.087 | 1.922 | 0.055 |

Null deviance: 213.66 on 262 degrees of freedom; Residual deviance: 178.39 on 257 degrees of freedom; AIC = 190.39; Number of Fisher Scoring iterations: 6; Obs: 27 observations deleted due to missingness.

**Figure S1**. Area under the receiver operating characteristic (ROC) curve calculated by DeLong method.


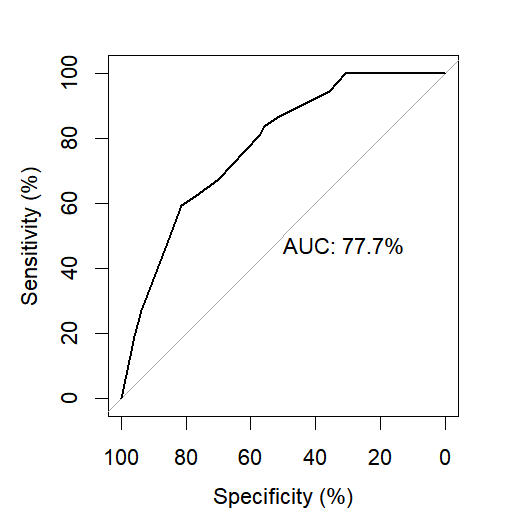


AUC = 0.777 (95% CI = 0.705-0.849)

**Table S2.** Logistic regression model results for factors associated with positivity for humans.

| Coefficient | Estimate | Std. Error | z value | Pr(>\|z\|) |
| --- | --- | --- | --- | --- |
| Intercept | -2.291 | 0.506 | -4.531 | < 0.001 |
| Gender: |  |  |  |  |
| Female | Reference |  |  |  |
| Male | 1.061 | 0.499 | 2.128 | 0.033 |
| Income range (minimum wage): |  |  |  |  |
| Less than 1 minimum wage | Reference |  |  |  |
| 1–3 minimum wages | -1.327 | 0.510 | -2.604 | 0.009 |
| More than 3 minimum wages | -0.769 | 1.119 | -0.687 | 0.492 |

Null deviance: 142.02 on 302 degrees of freedom; Residual deviance: 130.03 on 299 degrees of freedom; AIC: 138.03; Number of Fisher Scoring iterations: 6; Obs:1 observation deleted due to missingness.

**Figure S2.** Receiver operating characteristic (ROC) curve for *Mycoplasma* spp. positivity in humans, with area under the curve (AUC) and 95% confidence interval calculated using the DeLong method.


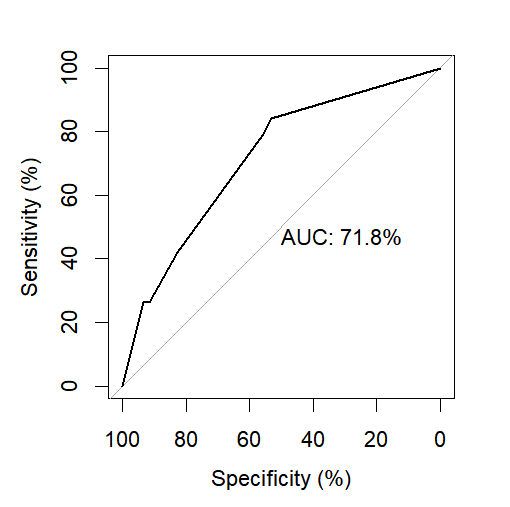


AUC = 0.718 (95% CI = 0.607-0.830)
